# Dietary Habits and Lifestyle Practices among University Students in Universiti Brunei Darussalam

**DOI:** 10.21315/mjms2018.25.3.6

**Published:** 2018-06-28

**Authors:** Tok Chen Yun, Siti Rohaiza Ahmad, David Koh Soo Quee

**Affiliations:** 1 PAPRSB Institute of Health Sciences, Universiti Brunei Darussalam; 2 Chair Professor of Occupational Health and Medicine, Universiti Brunei Darussalam

**Keywords:** obesity, eating habits, university students, physical exercise, transition

## Abstract

**Background:**

Young adults are at risk of developing obesity, especially when transitioning into university life as they become responsible for their daily eating and lifestyles. This study estimates the prevalence of overweight/obesity and explores the eating patterns and lifestyle practices of university students.

**Methods:**

A cross-sectional study was conducted at Universiti Brunei Darussalam (UBD). A total of 303 students participated. Data was collected from January to April 2016. Self-designed questionnaires comprised questions pertaining to current weight, self-reported height data, information on eating habits, exercise and knowledge of the food pyramid. The collected data were used to compare and contrast eating habits and lifestyle practices among overweight/obese students with those of non-overweight/obese students.

**Results:**

The prevalence of overweight/obesity was 28.8% (95% CI: 24.0%, 34.0%). The majority ate regular daily meals, but more than half skipped breakfast. Frequent snacking, fried food consumption at least three times per week and low intake of daily fruits and vegetables were common. The frequency of visits to fast food restaurants was significantly higher in the overweight/obese. 25.4% of the students exercised at least three times per week. Almost all students are aware of balanced nutrition and the food pyramid.

**Conclusions:**

Most university students had poor eating habits, although the majority had good nutrition knowledge. By way of recommendation, the university is encouraged to provide a multi-disciplinary team specialising in health promotion that includes nutrition and physical activity programmes to increase the awareness among the university students.

## Introduction

In 2005, a global burden of obesity stated that 33.0% of the adult population (1.3 billion people) is overweight/obese. It predicts that this percentage would likely increase to 57.8% (3.3 billion people) by the year 2030 if the trend persists (1). In Brunei Darussalam, the reported prevalence of obesity has increased from 12% in 1996 to 27.2% in 2011. This alarming rise has attracted the attention and concern of from the public because obesity is a recognised risk factor for numerous non-communicable diseases (NCDs) such as diabetes mellitus, hypertension, cardiovascular diseases and stroke (2).

The problems associated with obesity affect not only the adult population but also the youth. An overweight child or teenager is at a higher risk of being overweight/obese as an adult (3) and of developing adult diseases. Although the onset and development of obesity are most apparent during childhood (5), university students also undergo a critical period when their behaviours are conducive to change often resulting in weight gain (6). A study conducted in the International Medical University of Malaysia found that out of 240 clinical students, 72 were either overweight/obese (based on the World Health Organisation body mass index cut-offs for Asian population, i.e. BMI > 23.0 kg/m^2^) (7). The emerging practice of dieting for weight loss and image purposes among university students (8) and their effects on university students’ behaviours require public attention.

College weight gain is likely during the transition into university life, which is a critical period when young adults’ behaviours including dietary habits are conducive to change as they gain independence in making food choices (3, 4). These groups of individuals are at higher risk of developing unhealthy eating behaviours with inadequate nutrient intake, as shown by Gan et al. (5). Some of these behaviours include irregular meals, not eating breakfast, reduced fruit and vegetable intake and increased consumption of fried food (6). Apart from the change in dietary habits, poor exercising habits, bad time management and the increasing amount of stress from school work also contribute to weight gain (9).

Moreover, the opening of numerous fast food stores, cafés and restaurants provide university students more opportunities to dine outside instead of consuming self-prepared meals (10). The improper eating habits developed during this stage of life can continue into adulthood.

Studying the change in dietary habits and lifestyle practices among university students can help educate them on the importance of preventing early development of obesity by adopting healthy lifestyles. It is hoped that this study can increase the awareness of healthy lifestyle and eating among young adults, thereby reducing the risks of developing chronic diseases.

This research estimates the prevalence of overweight/obesity among students in a university in Brunei Darussalam, to compare eating habits and lifestyle practices of overweight/obese university students with that of non-overweight/obese university students and to explore students’ views about balanced nutrition, dieting and self-body image.

## Materials and Methods

### Study Design, Population and Sample

A cross-sectional study was conducted through self-administered questionnaires in Malay and English from February to March 2016 at the university. A total of seven faculties were involved:

- Faculty of Arts and Social Sciences (FASS)
- School of Business and Economics (SBE)
- Faculty of Science (FOS)
- Faculty of Integrated Technologies (FIT)
- Sultan Hassanal Bolkiah Institute of Education (SHBIE)
- PAPRSB Institute of Health Sciences (IHS)
- Academy of Brunei Studies (APB)

Data were collected from the students attending each faculty for three days. University students of all ages excluding international students were eligible for participation. Participation was voluntary with informed consent. A total of 303 university students participated in the study. The sample size was calculated using the following equation (11); where *n* is the minimum sample size required in the study, Z is the area under normal curve corresponding to the desired confidence interval used in this study, i.e. 95% CI (1.96), P is the prevalence of overweight/obese adult in Brunei Darussalam [(27.2%; Zakaria et al. (2)], and D is the precision [difference between sample mean and population mean (+/− 5%)].

$$\begin{array}{l} n=\text{Z}2[\text{P}(1-\text{P})]/\text{D}2\\ n=(1.96)2[(0.272)(0.728)]/(0.05)2\\ n=304 \end{array}$$

### Research Instruments

All research instruments, including the Participant Information Sheet (PIS), consent forms and questionnaires were available in Malay and English. The self-administered structured questionnaire (developed based on an adaptation from previous studies and literature search) consisted of 31 multiple choice questions (5, 6, 10, 12, 13). Apart from obtaining the sociodemographic information such as age, gender, ethnicity, current study semester, faculty, and accommodation status, the questions were designed to explore the eating patterns and lifestyles of university students. Personal views on dieting and self-body image were also solicited together with questions exploring their knowledge about balanced nutrition, eating patterns, lifestyles and daily exercises. Prior to conducting the research, to ensure the validity and reliability of the self-designed questionnaire, both English and Malay versions were tested on ten randomly selected university students to access their comprehensibility. No significant amendments were made based on the pre-test.

An electronic weighing scale (Brand/Model: Tanita/HD-382 Australia) was used for participants’ weight (in kilogram) measurement without heavy clothing (e.g. jackets) and accessories. The weight values along with self-reported height (in centimetres) values were used to calculate and classify body mass index [BMI] (overweight is a BMI greater than or equal to 25, and obesity is a BMI higher than or equal to 30) (11).

Definition of terms:

Snacking: refers to the intake of food between regular meals.
Regular exercise: refers to physical activities for at least 3–4 times per week.
Dieting: refers to restrictions in daily calorie consumption associated with unbalanced nutrient intake.
Knowledge of food pyramid: refers to an understanding of the main components (carbohydrate, protein, vitamins, fat and oil) of the food pyramid as well as the recommended daily portions.

Ethical approvals for this study were received from the Medical and Health Research Ethics Committee (MHREC), Ministry of Health, Brunei Darussalam and the Ethics Committee of PAPRSB Institute of Health Sciences (IHSREC), Universiti of Brunei Darussalam.

### Statistical Analysis

Data collected from the questionnaires were entered and analysed using IBM SPSS Statistics version 21.0 for Windows. The statistical analysis included the estimation of the proportion of university students who were overweight/obese with 95% confidence interval (CI) and chi-square test to compare eating habits and lifestyle practices of overweight/obese university students with that of non-overweight/obese university students.

## Results

### Demographic Data of Respondents

A total of 303 university students were recruited during the study period, of which 83 (27.4%) were male, and 220 (72.6%) were female. The response rate among those approached to participate was 95.3%, with a total of 15 rejections (no reason was given). Table 1 presents the sociodemographic data of the participants.

### Prevalence of Overweight/Obesity among University Students

The median body weight of the respondents was 56.3 kg (interquartile range (IQR) = 15.4 kg), and the mean self-report height was 1.60 metres (SD = 0.084). The median BMI was 22.5 kg/m2 (IQR = 5.8 kg/m2). The BMI category according to WHO (11), 58.1% university students was in the normal weight category, and 18.2% and 10.6% were overweight and obese, respectively (Table 2). The prevalence of overweight/obesity among the students was 28.8% (87 out of 303) (95% CI: 24.0%, 34.0%) (Table 2).

### Eating Habits of University Students

Out of 303 university students, 226 (74.6%) reported eating meals regularly on a daily basis with 42.6% (129 out of 303) practising consuming breakfast daily. The majority (52.5%) consumed three meals per day, while 101 (33.3%) of university students consumed less than three meals and 43 (14.2%) more than three meals. Many of the participants had a habit of snacking regularly and consumed fried food at least 3–5 times per week (82.2%, 60.7%, respectively). Only 23.4% (71 out of 303) and 9.2% (28 out of 303) of participants consumed vegetables and fruits every day, respectively, which is relatively low.

Table 3 compares eating habits between the non-overweight/obese populations with that of overweight/obese. The number of regular daily meals differed significantly between these two groups (*P* = 0.011).

### Lifestyle Practices of University Students

Most students (80.5%) would sometimes prepare/cook their meals, but very few (24.1%) would eat a variety of food (rice, meat, vegetables and fruits) as required for a balanced diet. The frequency of eating at restaurants, fast food stores, and cafés was relatively low, where many reported going to these places less than three times weekly (60.4%, 89.4%, 94.1%, respectively). However, all three practices were significantly (*P* < 0.05) associated with BMI status, where a higher proportion of nonoverweight/obese population (43.5%) eat out more frequently, while more of the overweight/obese population visited fast food stores (17.2%) and cafés (10.3%) more frequently (Table 4).

Most students (72.3%) ate with family at home at least three times weekly. As much as 58.7% (178 out of 303) of all participants preferred eating cheap food over healthy/nutritious food. This was significantly (*P =* 0.042) true for the overweight/obese population (67.8%). The majority (70.6%) ate more when feeling stressed. Regarding physical activity, 78.5% (238 out of 303) walked around the campus when going to classes. However, for the frequency of weekly exercise, only ten students (3.3%) exercised daily, while others exercised three to four times per week (22.1%), one to two times (36.6%), or rarely exercised (38.0%).

### Dieting, Balanced Nutrition and Self-Body Image

The majority of students were aware of the food pyramid (96.4%) and the concept of balanced nutrition (96.0%) (Table 5). Although the majority 82.5%) were concerned about body size and physical appearance, slightly less than half (47.9%) had tried dieting. The main reason for dieting (34.7%) (those who never dieted were questioned why they think other people dieted) was to be strong and healthy.

## Discussion

In this study, 28.8% of participants were overweight or obese, and 10.6% of the populations were obese. Although this percentage is lower when compared to the Bruneian adult obesity rate of 27.2% reported in the 2011 National Health and Nutritional Status Survey (2), the university obesity rate can still be worrying considering the younger age of participants.

The prevalence of overweight/obesity was similar among male and female students with a difference of only 0.3% (Table 2). This finding is different from the study conducted in the International Medical University of Malaysia (12), where male university students were more overweight/obese (15.3% more).

Eating regular meals with daily breakfast is considered healthy eating behaviour. Several studies concluded that the habit of skipping breakfast was associated with weight gain and a higher BMI value (13, 14). In this study, although most participants ate meals regularly, more than half did not eat breakfast daily. This result is similar to the findings of a Malaysian study (6) where 56.1% reported not consuming daily breakfast. It is possible that meal skipping caused frequent snacking as the majority of the participants admitted to snacking between regular meals. An association between daily meals frequency and BMI status was identified from our study where a higher proportion of overweight/obese participants (23.0%) consumed more than three meals daily (Table 3).

Hakim et al. (15) emphasised that skipping meals leads to more eating throughout the day including frequent snacking, which can subsequently result in weight gain. Minimal intake of daily fruits and vegetables combined with increased fried food consumption is common among university students (6, 12, 15). Such a trend was also observed among our participants (Table 3).

The phenomenon of nutrition transition is emerging globally in which diets are shifting away from home food intake to dependence on outdoor processed food that is high in fats, salt and sugar (16). The majority of our respondents prefer eating lunch in the campus cafeteria (51.1%) instead of bringing lunch from home (11.2%), indicating their reliance on outside food. Furthermore, they resorted to eating instant noodles when required to cook their meals, while few would eat a balanced meal including a variety of food (i.e., rice, meat, vegetables and fruits).

The frequency of visits to fast food restaurants and cafés were significantly higher in the overweight/obese population, suggesting consumption of more food that is high in fat, salt and sugar. Hakim et al. (15) believed that increasing accessibility to fast food stores is closely linked to overweight or obesity as there is an associated risk of consuming high energy food, sweetened drinks and fatty food but low intake of nutritious food. Fast food is a quick and cheap choice for university students, especially when the time is limited and there is a large university workload. The majority of the respondents preferred cheap food to healthy/nutritious food, especially among the overweight/obese population (*P* = 0.042).

It is important to keep a balance between energy intake and energy expenditure, as disruption of this balance can lead to obesity (14, 17). Physical activity is also an important determining factor of weight status. A combination of low physical activity with poor dietary habits increases the risk of overweight or obesity (17). In this study, most participants adopted the habit of walking around the campus, but only 25.4% (77 out of 303) of participants engaged in physical exercises at least three times per week. According to the WHO guidelines (18), physical activity of moderate intensity for at least 150 minutes throughout the week (equivalent to 30 min/day for five days) is recommended for ages 18–64 years. The majority of the participants did not meet these requirements.

Although some of the reported eating patterns were unhealthy, the majority of students had good knowledge of the food pyramid and balanced nutrition. Due to stress, heavy workload and lack of time, university students tend to make poor food choices (7). Hence, it is challenging for them to adhere to the food pyramid.

This study reported that female participants were more concerned about physical size and appearance, and slightly more females tried dieting compared to males. Similar results were also seen in a previous study (9), where being overweight was more of a fear among female students.

This study was subject to a number of limitations. As a list of the names of all attending students was not available, convenience sampling was used instead of random sampling, hence limiting the validity of the data. However, the response rate of those approached to participate in the study was high (95.3%). Although self-reported height values could be under- or overestimated by participants, some studies (19, 20) had shown that BMI calculations based on self-reported data were still able to classify most of the population into the correct BMI categories.

The BMI classification used in this study was based on the WHO international cut-off values. However, considering the WHO Asian BMI cut-off values, the prevalence of overweight/obesity among students may be underestimated.

In regard to the questionnaires, no quantitative data (such as daily food portions, calorie intake, and duration of daily exercises) was available to identify the association between the lifestyle practices and BMI status of university students. In addition, the type of food and snacks that university students tend to eat on a daily basis were not identified. Psychological factors associated with overweight/obesity leading to students’ desire for weight loss practices were also not explored.

## Conclusion

The prevalence of overweight/obesity among this population of university students was 28.8% and affected males and females equally (28.9% versus 28.6%). A higher proportion of females were concerned about body size and physical appearance; hence dieting was more common among them. Although most university students reported having good knowledge of the food pyramid and balanced nutrition, the majority did not adhere to and practiced such healthy eating habits. Most of them skipped breakfast, snacked frequently, consumed fried food often and had a low intake of daily fruits and vegetables.

The transition from home food to increased reliance on outside food such as fast food common among the respondents especially among the overweight/obese population. Physical activity was low among students and less than WHO recommended levels. Therefore, the university should provide a multi-disciplinary team to support nutrition and physical activity programmes to increase the awareness among the university students (21). Physical activity programmes in the campus may have a positive impact on the student’s behaviour towards exercise.

## Figures and Tables

**Table 1:** Sociodemographic characteristics of respondents (*n* = 303)

Characteristics	Median (IQR)	*n* (%)
Gender		
Male		83 (27.4)
Female		220 (72.6)
Age (year)	20.0 (2)^a^	
Ethnicity		
Malay		221 (72.9)
Chinese		65 (21.5)
Others		17 (5.6)
Faculty		
Faculty of Arts and Social Sciences (FASS)		55 (18.2)
School of Business and Economics (SBE)		60 (19.8)
Sultan Hassanal Bolkiah Institute of Education (SHBIE)		25 (8.3)
Academy of Brunei Studies (APB)		21 (6.9)
Faculty of Integrated Technologies (FIT)		32 (10.6)
Faculty of Science (FOS)		56 (18.5)
Institute of Health Sciences (IHS)		54 (17.8)
Accommodation status		
On campus		57 (18.8)
Off campus		246 (81.2)
Year of studying		
Year 1		135 (44.6)
Year 2		149 (49.2)
Year 3		8 (2.6)
Year 4		11 (3.6)

IQR = Interquartile range

a The distribution is skewed to the right

**Table 2:** Distribution of students according to BMI status

WHO International BMI Classifications				

BMI (kg/m^2^)	BMI category	Male *n* = 83 (%)	Female *n* = 220 (%)	Total *n* = 303 (%)
< 18.5	Underweight	14 (16.9)	26 (11.8)	40 (13.2)
18.5–24.9	Normal	45 (54.2)	131 (59.5)	176 (58.1)
25–29.9	Overweight	14 (16.9)	41 (18.6)	55 (18.2)
≥ 30.0	Obese	10 (12.0)	22 (10.0)	32 (10.6)

**Table 3:** Factors (eating habits) associated with overweight/obesity

Variable	*n* = 303 (%)	Non-overweight/obese *n* = 216 (%)	Overweight/obese *n* = 87 (%)	*x^2^*statistics^a^ (df)	*P*-value^a^
Eat meals regularly on daily basis					
Yes	226 (74.6)	165 (76.4)	61 (70.1)	1.29 (1)	0.256
No	77 (25.4)	51 (23.6)	26 (29.9)		
Number of regular meals					
< 3 meals/day	101 (33.3)	71 (32.9)	30 (34.5)	9.00 (2)	0.011
3 meals/day	159 (52.5)	122 (56.5)	37 (42.5)		
> 3 meals/day	43 (14.2)	23 (10.6)	20 (23.0)		
Eat breakfast every day					
Yes	129 (42.6)	95 (44.0)	34 (39.1)	0.61 (1)	0.435
No	174 (57.4)	121 (56.0)	53 (60.9)		
Snack in between regular meals					
Yes	249 (82.2)	183 (84.7)	66 (75.9)	3.32 (1)	0.068
No	54 (17.8)	33 (15.3)	21 (24.1)		
How often do you eat vegetables					
Everyday	71 (23.4)	52 (24.11)	19 (21.8)	0.24 (2)	0.886
3–5 times per week	130 (42.9)	91 (42.1)	39 (44.8)		
Rarely	102 (33.7)	73 (33.8)	29 (33.3)		
How often do you eat fruits					
Everyday	28 (9.2)	24 (11.1)	4 (4.6)	3.26 (2)	0.196
3–5 times per week	127 (41.9)	90 (41.7)	37 (42.5)		
Rarely	148 (48.8)	102 (47.2)	46 (52.9)		
How often do you fried food					
Everyday	62 (20.5)	47 (21.8)	15 (17.2)	3.62 (2)	0.164
3–5 times per week	184 (60.7)	124 (57.4)	60 (69.0)		
Rarely	57 (18.8)	45 (20.8)	12 (13.8)		
Daily water intake					
< 2 L	128 (42.2)	88 (40.7)	40 (46.0)		
≥2 L	175 (57.8)	128 (59.3)	47 (54.0)	0.70 (1)	0.404

**Table 4:** Factors (lifestyle practices) associated with overweight/obesity

Variable	*n* = 303 (%)	Non-overweight/obese *n* = 216 (%)	Overweight/obese *n* = 87 (%)	*x^2^*statistics^a^ (df)	*P*-value^a^
Do you bring lunch to school					
Yes	34 (11.2)	21 (9.7)	13 (14.9)	1.70 (1)	0.193
No	269 (88.8)	195 (90.3)	74 (85.1)		
Where do you get lunch on a typical school day					
I don’t eat my lunch	15 (5.5)	12 (6.1)	3 (4.1)	1.86 (3)	0.601
I go home during lunch times	45 (16.5)	30 (15.2)	15 (20.3)		
I eat/buy lunch at campus cafeteria	139 (51.1)	100 (50.5)	39 (52.7)		
I eat lunch at restaurants in town	73 (26.8)	56 (28.3)	7 (23.0)		
Do you prepare/cook your own meals					
Always	26 (8.6)	21 (9.7)	5 (5.7)	1.78 (2)	0.412
Sometimes	244 (80.5)	170 (78.7)	74 (85.1)		
Never	33 (10.9)	25 (11.6)	8 (9.2)		
What do you usually eat when you had to prepare/cook your own meals					
Rice, meat, vegetables and fruits	73 (24.1)	55 (25.5)	18 (20.7)	2.46 (3)	0.483
Rice and meat/vegetables	104 (34.3)	71 (32.9)	33 (37.9)		
Meat/vegetables/Fruits only	22 ( 7.3)	18 ( 8.3)	4 ( 4.6)		
Instant noodles/any noodles	104 (34.3)	72 (33.3)	32 (36.8)		
How often do you eat out in restaurants					
< 3 times per week	183 (60.4)	122 (56.5)	61 (70.1)	4.82 (1)	0.028
3 or more times per week	120 (39.6)	94 (43.5)	26 (29.9)		
How often do you eat fast foods (McD, KFC, Jollibee, Sugarbun, Burger King)					
< 3 times per week	271 (89.4)	199 (92.1)	72 (82.8)	5.77 (1)	0.016
3 or more times per week	32 (10.6)	17 ( 7.9)	15 (17.2)		
How often do you visit cafés (Starbucks, Coffee Bean, Gloria Jeans)					
< 3 times per week	285 (94.1)	207 (95.8)	78 (89.7)	4.24 (1)	0.040
3 or more times per week	18 ( 5.9)	9 ( 4.2)	9 (10.3)		
How often do you order food takeaways/deliveries					
< 3 times per week	232 (76.6)	167 (77.3)	65 (74.7)	0.23 (1)	0.629
3 or more times per week	71 (23.4)	49 (22.7)	22 (25.3)		
How often do you eat with your family at home					
< 3 times per week	84 (27.7)	58 (26.9)	26 (29.9)	0.23 (1)	0.629
3 or more times per week	219 (72.3)	158 (73.1)	61 (70.1)		
Do you choose food that cost less over healthy/nutritious food or the other way round					
Healthy/nutritious food	125 (41.3)	97 (44.9)	28 (32.2)	4.14 (1)	0.042
Food that costs less	178 (58.7)	119 (55.1)	59 (67.8)		
Do you eat more when feeling stressed					
Yes	214 (70.6)	152 (70.4)	62 (71.3)	0.02 (1)	0.877
No	89 (29.4)	64 (29.6)	25 (28.7)		
How often do you exercise					
Everyday	10 (3.3)	8 (3.7)	2 (2.3)	0.68 (3)	0.877
3–4 times per week	67 (22.1)	49 (22.7)	18 (20.7)		
Once or twice per week	111 (36.6)	77 (35.6)	34 (39.1)		
Rarely	115 (38.0)	82 (38.0)	33 (37.9)		
Do you walk around campus					
Yes	238 (78.5)	171 (79.2)	67 (77.0)	0.17 (1)	0.679
No	65 (21.5)	45 (20.8)	20 (23.0)		

a Chi-square statistical analysis with significance at *P* < 0.05

**Table 5:** Knowledge and views on dieting, balanced nutrition and self-body image by gender

Variable	*n* = 303 (%)	Male *n* = 83 (%)	Female *n* = 220 (%)
Do you know the ‘Food Pyramid’			
Yes	292 (96.4)	78 (94.0)	214 (97.3)
No	11 (3.6)	5 (6.0)	6 (2.7)
What is a balanced nutrition			
A diet consisting of mainly meat	2 (0.7)	0 (0.0)	2 (0.9)
A diet consisting of mainly fruits and vegetables	8 (2.6)	2 (2.4)	6 (2.7)
A diet consisting of meat, vegetables and other variety of foods	291 (96.0)	80 (96.4)	211 (95.9)
Others	2 (0.7)	1 (1.2)	1 (0.7)
Are you concerned about your body size and physical appearance			
Yes	250 (82.5)	66 (79.5)	184 (83.6)
No	53 (17.5)	17 (20.5)	36 (16.4)
Have you tried dieting before			
Yes	145 (47.9)	34 (41.0)	111 (50.5)
No	158 (52.1)	49 (59.0)	109 (49.5)
Reasons for dieting			
To be strong and healthy	105 (34.7)	36 (43.4)	69 (31.4)
To be slim/thin and confident	90 (29.7)	19 (22.9)	71 (32.3)
To look beautiful in clothes	24 (7.9)	9 (10.8)	15 (6.8)
To maintain my weight	84 (27.7)	19 (22.9)	65 (29.5)
